# *In Vivo* Evaluation of Sepigel-Based Meglumine Antimoniate and Amphotericin B for Cutaneous Leishmaniasis Treatment

**DOI:** 10.3390/pathogens13080712

**Published:** 2024-08-22

**Authors:** Atteneri López-Arencibia, Carlos J. Bethencourt-Estrella, Diana Berenguer, Angélica Domínguez-de-Barros, M. Magdalena Alcover, Marcella Sessa, Lyda Halbaut, Roser Fisa, Ana Cristina Calpena-Campmany, A. Elizabeth Córdoba-Lanús, Jacob Lorenzo-Morales, Cristina Riera, José E. Piñero

**Affiliations:** 1 Instituto Universitario de Enfermedades Tropicales y Salud Pública de Canarias (IUETSPC), Universidad de La Laguna (ULL), Avenida Astrofísico Francisco Sánchez s/n, 38206 La Laguna, Tenerife, Spain; cbethene@ull.edu.es (C.J.B.-E.); angelica4arealejos@gmail.com (A.D.-d.-B.); acordoba@ull.edu.es (A.E.C.-L.); jmlorenz@ull.edu.es (J.L.-M.); jpinero@ull.edu.es (J.E.P.); 2 Consorcio Centro de Investigación Biomédica en Red M.P. de Enfermedades Infecciosas (CIBERINFEC), Instituto de Salud Carlos III, 28006 Madrid, Spain; 3 Departamento de Obstetricia y Ginecología, Pediatría, Medicina Preventiva y Salud Pública, Toxicología, Medicina Legal y Forense y Parasitología, Universidad de La Laguna (ULL), 38200 Santa Cruz de Tenerife, Tenerife, Spain; 4 Department of Biology, Health and Environment, Laboratory of Parasitology, Faculty of Pharmacy and Food Sciences, University of Barcelona, 08028 Barcelona, Spainmmagdalenaalcoveramengual@ub.edu (M.M.A.); rfisa@ub.edu (R.F.); 5 Department of Pharmaceutical Technology and Physicochemistry, Faculty of Pharmacy and Food Sciences, University of Barcelona, 08028 Barcelona, Spain; marcellass93@gmail.com (M.S.); halbaut@ub.edu (L.H.); anacalpena@ub.edu (A.C.C.-C.)

**Keywords:** *Leishmania amazonensis*, *in vivo*, meglumine antimoniate, amphotericin B, Sepigel

## Abstract

Cutaneous leishmaniasis (CL) poses a significant public health concern in endemic regions due to its increasing prevalence and substantial impact on affected individuals. This disease is primarily caused by the *Leishmania* protozoa, which are transmitted through insect bites, and it manifests as a range of symptoms, from self-healing lesions to severe disfigurement. Current treatments, which often involve the parenteral administration of antimonials, face challenges such as poor compliance and adverse effects. This study investigates the efficacy of topical formulations containing meglumine antimoniate (MA) and amphotericin B (AmB), using Sepigel as an excipient, for treating CL. In the *in vivo* study, BALB/c mice infected with *L. amazonensis* developed lesions at the injection site five weeks post-infection. Subsequently, the mice were divided into eight groups: untreated mice, mice treated orally with miltefosine, mice treated intraperitoneally with MA, and mice treated topically with 15%, 22.5%, and 30% MA-Sepigel, as well as those treated with AmB-Sepigel. Treatments were applied daily for two weeks, and the results revealed a significant reduction in lesion size and parasite burden following topical application, particularly with the AmB-Sepigel formulations and 30% MA-Sepigel. Additionally, Sepigel-based treatments demonstrated improved patient compliance and reduced toxicity compared to systemic therapies. These findings underscore the potential of Sepigel-based formulations as a promising alternative for CL treatment. They offer enhanced efficacy and tolerability, while reducing the systemic toxicity associated with conventional therapies.

## 1. Introduction

Leishmaniasis is an infectious disease caused by the protozoan *Leishmania*, which is transmitted through mammals through the bites of female insects of the genus *Phlebotomus*. This disease encompasses a variety of forms that pose serious public health problems in endemic regions across 98 countries. The three main forms are visceral leishmaniasis (VL), mucocutaneous leishmaniasis (MCL), and cutaneous leishmaniasis (CL), with CL being the most common [1]. Leishmaniasis manifests as multifactorial diseases influenced by parasite and its reservoirs, vectors, and the human immune system. Ecosystem changes, such as climatic shifts, can also affect parasite infectivity. For example, desert expansion can create conducive environments for the proliferation of vectors and reservoirs of CL parasites [2]. The prevalence of CL is rising in endemic areas due to favorable natural environmental changes, exacerbated by human activities such as urbanization, deforestation, regional conflicts, and migration [3]. CL presents a wide spectrum of symptoms, from self-healing lesions to painful open sores, and can cause significant skin disfigurement and disability. Healed ulcers often leave permanent scars, which can have profound psychological effects on patients, leading to depression, anxiety, and a diminished quality of life. Moreover, complications from concurrent HIV infection and leishmaniasis present formidable challenges, intensifying the severity of both conditions and have been documented in numerous countries worldwide [4].

The limited availability of drugs in endemic areas is due to the fact that most treatment regimens require parenteral administration, leading to severe patient discomfort, low compliance, and poorer therapeutic outcomes. The standard treatment for CL is intralesional or parenteral administration of pentavalent antimonials, but the most common adverse effects include fatigue, leukopenia, thrombocytopenia, and cardiotoxicity [5]. Intralesional injections are painful and require 5–8 treatment sessions. Often, topical treatments are combined with parenteral administration to enhance therapeutic efficacy. Several drugs, both new and existing, have been reformulated into topical dosage forms and are currently being studied to expand the therapeutic options for CL [6]. Miltefosine, presented as the first oral drug for this parasitosis, has emerged as a good alternative, though its use is not yet licensed in many countries. While various drugs are available as alternatives, they generally remain ineffective, costly, and protracted, and they frequently produce side effects and cause resistance [7]. Despite the significant challenges presented by neglected tropical diseases (NTDs), these conditions are inadequately researched and discussed, consequently, the World Health Organization (WHO) strongly advocates for increased efforts to combat and eliminate NTDs [8].

Gels are used in topical pharmaceutical formulations because of their ability to control drug release, and these include a gelling agent along with solubilizers. The main challenge of gels is to maintain the drug in a solubilized state while ensuring controlled release. Recent research is studying gels for the topical treatment of skin cancer, with the aim of improving drug penetration, retention and efficacy, while minimizing systemic side effects [9]. Sepigel 305, a liquid polymer used as a gelling agent in cosmetics, is pre-neutralized and works effectively over a wide pH spectrum. It serves not only as a thickener, but also as an exceptional stabilizer and texturizing agent, and the resulting gels have a medium consistency and opalescence, providing a refreshing, evanescent quality with an optimal dermo-cosmetic appearance [10].

MA and AmB are both widely used in the treatment of leishmaniasis. MA has been used for many years and is often the first choice for treatment. However, its use can lead to complications due to side effects, as it is typically administered intramuscularly or intralesionally for the treatment of VL or CL, respectively [11]. AmB, which interacts with ergosterol in the parasite’s cell membrane, is often considered the second choice for treatment. This is typically the case when there is no response to MA or when its use is contraindicated. The effectiveness of these drugs can vary depending on the patient’s immune status and the specific species of *Leishmania* involved [12]. The leishmanicidal activity of these compounds, along with their ability to induce apoptotic programmed cell death in *L. amazonensis*, has been demonstrated and confirmed [13].

**Figure 1:**
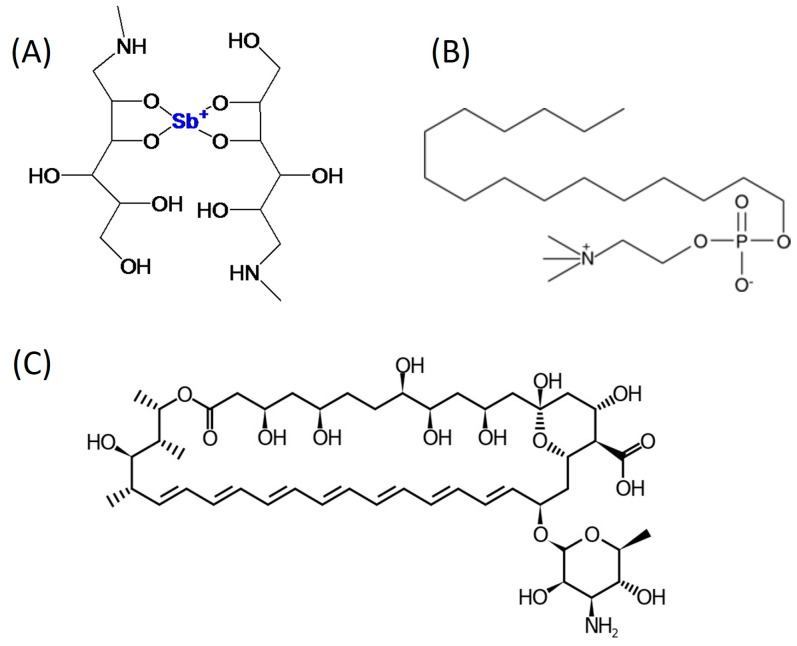
Chemical structure of MA (**A**), miltefosine (**B**), and AmB (**C**).

The current study evaluates the potential of topically applied gel formulations of MA and AmB as treatments for CL (Figure 1). Both MA and AmB gels have been previously characterized [14,15], with various physicochemical parameters assessed. These parameters include drug content stability over time, which was monitored under different conditions (room temperature, 4 °C, and 37 °C for 2 and 6 months for the AmB gel and MA gel, respectively). Additionally, the permeation and retention of the main active ingredients were examined using an *ex vivo* model with human skin, and the gels’ tolerance was tested on 10 healthy volunteers. Furthermore, cytotoxic effects and leishmanicidal activity were evaluated *in vitro* on promastigotes and intracellular amastigotes of *L. infantum*, showing positive results.

## 2. Materials and Methods

### 2.1. Chemicals

MA was sourced from Acros Organics (Thermo Fisher Scientific, MA, USA). AmB and Sepigel 305^®^ Seppic were obtained from Acofarma (Barcelona, Spain). Gentamicin, Dimethyl sulfoxide (DMSO), and NaOH came from Sigma-Aldrich (Darmstadt, Germany). TranscutolP^®^ was provided by Gattefossé (Barcelona, Spain). Miltefosine was purchased from Sigma-Aldrich (Merck, Madrid, Spain). The distilled water used for the assays was acquired from a Mili-Q^®^ Plus System (Millipore Co., Burlington, MA, USA).

### 2.2. Parasites

*Leishmania amazonensis* promastigotes (MHOM/BR/77/LTB0016) were cultured in Schneider medium Sigma-Aldrich (Darmstadt, Germany) supplemented with 10% fetal bovine serum (FBS; GIBCO) and 10 μg/mL of gentamicin (Sigma, Spain) at 26 °C. The parasite culture was monitored daily with an image-based cytometer Tali^®^ (Invitrogen by Life Technologies, Madrid, Spain). *In vitro* passages were conducted upon reaching the stationary growth phase, with the parasites’ infectivity sustained through passages in BALB/c mice.

### 2.3. Animals

Female BALB/c mice (*Mus musculus*) aged from 6 to 12 weeks were bred and maintained at the animal facilities of Universidad de La Laguna, San Cristóbal de La Laguna, Tenerife, Spain. The mice were housed in rooms controlled for temperature (22 ± 2 °C) and humidity (55 ± 10%), with continuous air renewal and a 12:12 light cycle. They were provided with a balanced diet for rodents and had unrestricted access to water. The protocol was approved by the Research Ethics and Animal Welfare Committee of the Universidad de La Laguna, in accordance with European Guidelines [16].

### 2.4. Gel Formulations

For the *in vivo* treatment, MA-based gel formulations were created using MA concentrations of 15%, 22.5%, and 30% (*w*/*v*) in Sepigel 305^®^. Initially, the active compound was measured and dissolved in DMSO to a maximum volume of 1% to ensure even distribution. Subsequently, Sepigel 305^®^ was added with continuous stirring to achieve a uniform gel formulation. The resulting gels were then stored at 4 °C until needed.

A fourth gel formulation was prepared using AmB. First, 30 mL of 1% Sepigel 305 gel base was prepared under stirring. Then, 150 mg of AmB was dissolved in 5 mL of DMSO, and 1 mL of this solution was added slowly under continuous stirring to the initial preparation. The final gel formulation was adjusted to pH 6 with a 2N NaOH solution, resulting in a final concentration of 1000 µg/mL of AmB [15].

### 2.5. In Vivo Infection and Treatment Administration

**Figure 2:**
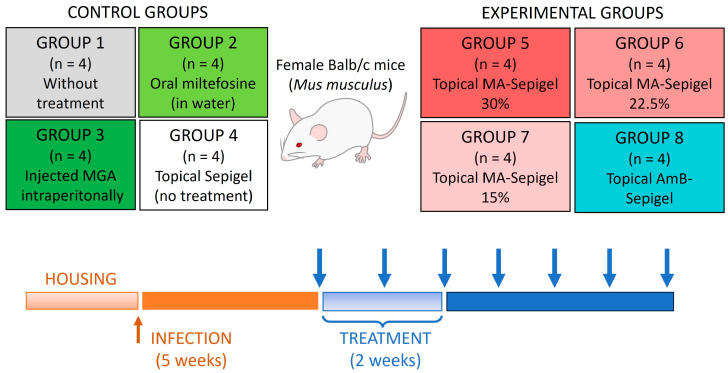
Scheme of treatment procedures used for the *in vivo* experiments. Blue arrows indicate the timeline of lesion measurement.

Female BALB/c mice (5–7 weeks old, weighing approximately 20 g) were infected at the base of the tail with 1 × 10^6^ stationary-phase *L. amazonensis* promastigotes in 50 µL of physiological saline solution. After a 5-week infection period, the mice underwent a 2-week daily treatment regimen. They received 50 µL of the various Sepigel formulations topically, covering the entire lesion/ulcer/inflammation. These included formulations with 15%, 22.5%, and 30% MA, just Sepigel, or AmB-Sepigel. Mice treated orally with miltefosine received the drug dissolved in their drinking water, with the dosage adjusted based on average water intake and the mice’s weight [17]. Mice treated with MA by intraperitoneal injection received 120 mg/kg/day. Given the average weight of the mice was 25 mg, the dosage was 3 mg/day per mouse, administered with insulin syringes of 0.3 mm diameter [18]. Lesion development was monitored weekly using a caliper, and lesion size was calculated by measuring the thickness and length. One month after the treatment concluded, the mice were sacrificed, and tissue samples from the skin (lesion) and liver were collected for parasite quantification. The scheme carried out in the *in vivo* study is shown in Figure 2.

### 2.6. DNA Extraction

Following the sacrifice of the mice, both treated and untreated, with various Sepigel formulations or injected treatments, skin and liver figure biopsy punch, and the liver tissues were extracted and immediately frozen. The biopsy specimens were then weighed and homogenized using a TissueRuptor (Qiagen, Hilden, Germany). This was followed by lysis with proteinase K and DNA isolation using the Illustra TM Tissue & Cells GenomicPrep Mini Spin Kit (GE Healthcare, Chicago, IL, USA).

### 2.7. Parasite Quantification

Quantification of parasites was conducted using qPCR. In each reaction, 2 µL of DNA extract samples (0.5 ng/µL) were amplified using 5 µL of Power SYBR Green PCR Master Mix (Applied Biosystems, Waltham, MA, USA) and 5 µM of each primer, resulting in a total volume of 10 µL. The qPCR reaction included 40 cycles, with the following stages: 20 s at 95 °C, 20 s at 60 °C, and 20 s at 72 °C. The final melting curve step was configured as 15 s at 95 °C, 1 min at 60 °C, and 15 s at 95 °C, using a Step One Plus real-time PCR machine (ThermoFisher Scientific, Waltham, MA, USA). The primer pair used was LMi-amaF–AAAATGAGTGCAGAAACCC and MLR–CGGCCCTATTTTACACCAACC′, targeting a fragment of the kDNA (qPCR-ama) (adapted from [19]).

A standard curve was established using serially diluted known concentrations (10 ng, 1 ng, 0.1 ng, 0.01 ng, 0.001 ng) of *L. amazonensis* DNA equivalents per reaction (MHOM/BR/77/LTB0016) (slope = 3.265; R^2^ = 0.98; E = 102.4%). All clinical tissues and standard samples were run in duplicate. A sample with a known quantity of *Leishmania* parasites served as the positive control.

To determine the number of *Leishmania* parasites in each tissue sample relative to the standard curve, the calculation was as follows: [parasite DNA equivalents per reaction/amount of tissue DNA per reaction] × 10^3^, expressed as the number of *Leishmania* parasites per mg of tissue DNA. Given the size of the *L. amazonensis* haploid genome, 83.15 fg of DNA was considered equivalent to one parasite.

### 2.8. Statistical Analysis

Results are presented as mean ± standard deviation (SD), based on a minimum of three independent experiments to ensure consistency. First, a Shapiro–Wilk test was performed to check the normality of the data (all groups were normally distributed, with *p* values between *p* = 0.105 and *p* = 0.618), and once this was corroborated, statistical differences between means were evaluated using one-way analysis of variance (ANOVA) for three or more samples, with Tukey’s test for pairwise comparisons of means. These analyses were performed using SigmaPlot 12.0 software (Systat Software, San Jose, CA, USA), with a significance level of *p* < 0.05 applied to all analyses.

## 3. Results

### 3.1. In Vivo Evaluation of Antileishmanial Activity of Experimental Sepigel Formulations and Control Groups in BALB/c Mice Infected by L. amazonensis

To evaluate the *in vivo* effects of Sepigel-based treatments on CL, BALB/c mice were infected with *L. amazonensis* in the dermis at the base of the tail. Starting five weeks after infection, the mice were treated daily for two weeks with four different treatments: topical MA-Sepigel at concentrations of 15%, 22.5%, and 30%, and AmB-Sepigel (Figure 2). The control groups included untreated mice, mice treated orally with miltefosine, and mice treated intraperitoneally with MA. Lesion development was monitored weekly.

**Figure 3:**
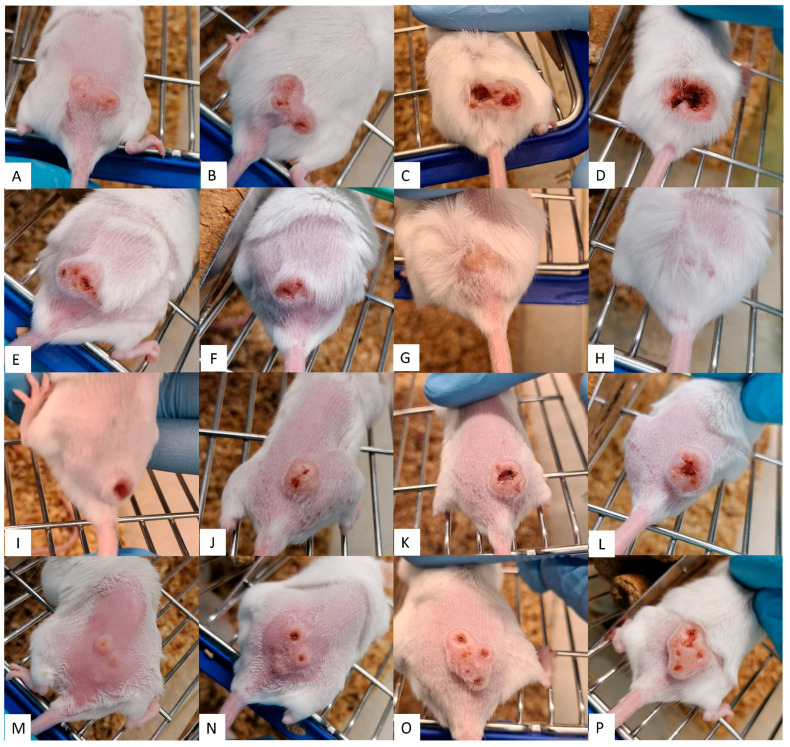
Efficacy of control treatments on CL lesions. Each row corresponds to a different treatment along the time. (**A**–**D**) without treatment; (**E**–**H**) oral miltefosine; (**I**–**L**) intraperitoneal MA; (**M**–**P**) topical Sepigel. Each column corresponds to a point in time: (**A**,**E**,**I**,**M**) shows the dimension of the CL lesions before treatment; (**B**,**F**,**J**,**N**) shows the dimension of the CL lesions after treatment (week 2); (**C**,**G**,**K**,**O**) shows the dimension of the CL lesions two weeks after treatment (week 4); (**D**,**H**,**L**,**P**) shows the dimension of the CL lesions four weeks after treatment (week 6).

**Figure 4:**
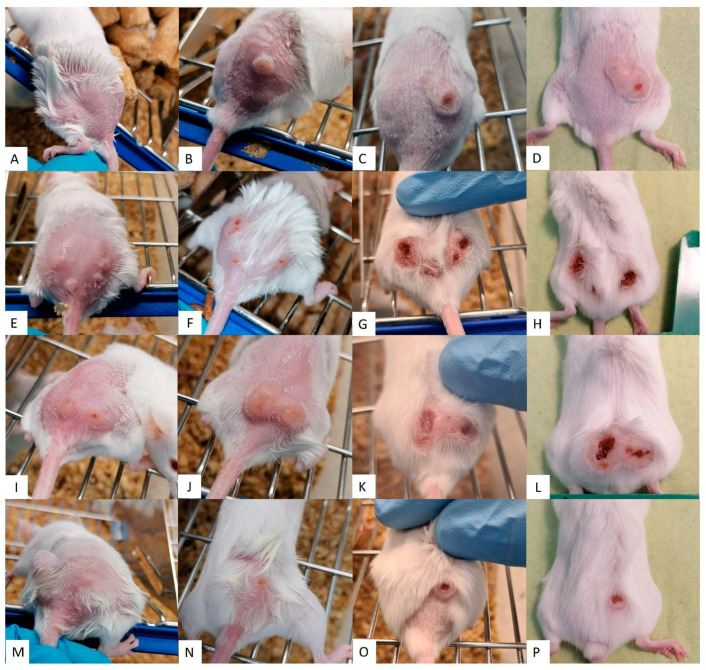
Efficacy of Sepigel-treatments on CL lesions. Each row corresponds to a different treatment along the time. (**A**–**D**) 15% MA-Sepigel; (**E**–**H**) 22.5% MA-Sepigel; (**I**–**L**) 30% MA-Sepigel; (**M**–**P**) AmB-Sepigel. Each column corresponds to a point in time. (**A**,**E**,**I**,**M**) shows the dimension of the CL lesions before treatment; (**B**,**F**,**J**,**N**) shows the dimension of the CL lesions after treatment (week 2); (**C**,**G**,**K**,**O**) shows the dimension of the CL lesions two weeks after treatment (week 4); (**D**,**H**,**L**,**P**) shows the dimension of the CL lesions four weeks after treatment (week 6).

The results from lesions in untreated controls, seen in Figure 3A–D, increased in size and inflammation and even developed ulcerations. However, treatments using Sepigel as an excipient (Figure 4), both MA and AmB, showed a marked decrease in lesion inflammation. Furthermore, the ulcerations in lesions treated with Sepigel-based formulations were much smaller than those in the untreated group. Interestingly, the lesions in mice treated orally with miltefosine (Figure 3E–H) were less inflamed and, while they did develop small ulcerations, these healed within four weeks after treatment. In addition, treatment with intraperitoneal MA (Figure 3I–L) appears to maintain the size of the lesion, at least after treatment, slightly decreasing its inflammation and ulceration.

In contrast, Figure 3 and Figure 4 showed that the lesion growth rate slowed significantly over the course of the Sepigel-based treatments, particularly two weeks post-treatment (Figure 4C,G,K,O). Notably, the mice treated with the highest concentration of MA exhibited the most pronounced response (Figure 4I–L). The AmB treatment (Figure 4M–P) also significantly reduced lesion size compared to untreated mice (Figure 3A–D), displaying even better results than those treated with MA-Sepigel (Figure 4D,H,L) four weeks post-treatment.

### 3.2. Evaluation of Sepigel-Based Treatments in the Evolution of Skin Lesions in Mice Infected with L. amazonensis

**Figure 5:**
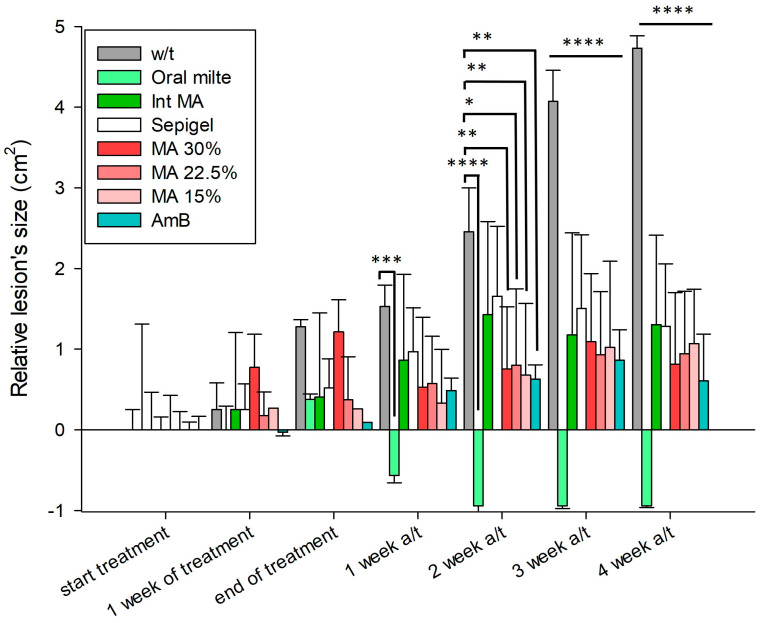
Progression of lesion size in *L. amazonensis* infected BALB/c mice treated with different concentrations of topical Sepigel-based treatments. Lesion size was measured in two dimensions using calipers, and the mean lesion diameters were determined. Lesions were treated topically with 50 µL of the formulations once daily for 2 weeks. Lesion size was recorded during treatment and up to 4 weeks after the end of treatment. w/t: without treatment; Oral milte: oral miltefosine; Int MA: intraperitoneally injected MA; MA: meglumine antimoniate; AmB: amphotericin B. a/t: after treatment. N = 4. * *p* < 0.05; ** *p* < 0.01; *** *p* < 0.001; **** *p* < 0.0001. All treatments evaluated at 3 weeks a/t and 4 weeks a/t showed *p* < 0.0001 with respect to the untreated control.

The evolution of lesion size in mice infected with *L. amazonensis* was monitored from the first day of treatment until four weeks after the treatment ended. Lesion size, representing the average area of the lesion in cm^2^, was recorded for four mice per group and the results are shown in Figure 5. The mean lesion sizes at the start of treatment were set to zero to facilitate comparison between groups. Thus, lesion sizes could increase, primarily in the untreated group, or decrease in groups receiving effective treatments.

Animals treated with AmB exhibited a decrease in lesion volume compared to the untreated control group. This reduction was most pronounced four weeks post-treatment, showing the most substantial decrease among all experimental groups. Notably, two weeks after treatment ended, the AmB-treated group began showing a marked decrease in lesion area compared to the untreated group. The group treated with miltefosine displayed an even more significant reduction from the untreated group in terms of lesion size, improving upon the initial measurements.

Regarding the MA treatments, results indicated a divergence from the untreated group from the first week post-treatment, with similar behavior observed across the three tested concentrations (30%, 22.5%, and 15%). However, only the group treated with the highest concentration (30%) exhibited a minor difference at the end of treatment; lesion measurements were initially similar to those of the untreated control, but subsequently, their lesion sizes decreased and surpassed those of the lower concentrations four weeks post-treatment, achieving the smallest lesion sizes of all groups. We can observe how Sepigel only seems to slightly decrease the size of the lesions, as its values resemble those of intraperitoneal MA, this may be associated with a mild anti-inflammatory effect related to the properties of the gel, as well as protection of the ulcer.

It is notable how the lesion sizes in the untreated control group continued to increase throughout the experiment, with exponential growth observed between the second and third weeks post-treatment. This growth corresponds with the ulcer developments and images documented in the previous section.

### 3.3. Evaluation of Parasite Burden in Treated Mice by Quantitative PCR

**Figure 6:**
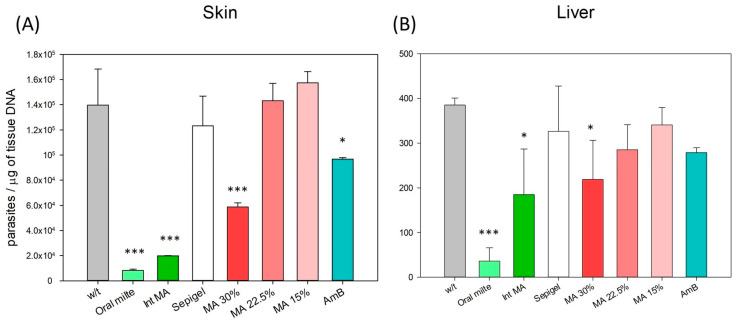
Skin and liver parasite burden in BALB/c mice from control and experimental groups. The number of *L. amazonensis* was quantified using qPCR from tissue of (**A**) skin (lesion edge) and (**B**) liver tissue from the different groups of mice at week 4 after the end of treatment. w/t: without treatment; Oral milte: oral miltefosine; Inj MA: intraperitoneally injected MA; MA: meglumine antimoniate in Sepigel; AmB: amphotericin B in Sepigel. N = 4. * *p* < 0.05; *** *p* < 0.001.

To assess the efficacy of MA and AmB Sepigel-based formulations against *L. amazonensis*, the parasite load in the skin (lesion edge) and liver was evaluated and represented in Figure 6. Animals treated with 30% MA-Sepigel and AmB-Sepigel demonstrated a significant reduction in parasite burden in the lesion compared to the untreated control group. For the two tissues studied—skin and liver of *L. amazonensis*-infected mice—those treated with MA showed a positive concentration–effect relationship. Specifically, the higher concentration of MA (30%) had a more substantial effect in reducing the parasite load in both tissues compared to the lower concentrations (22.5% and 15%). This indicates that a higher dose results in a greater leishmanicidal effect, corroborating observations from studies on lesion sizes and appearances. As can be seen in the graph, Sepigel alone did not affect the parasite load, as its values, although somewhat lower in the skin, resembled the values of the untreated control.

The data illustrate significant reductions in parasite load in the skin (lesion) when compared to the untreated control (*p* < 0.001 for MA 30%, and *p* = 0.013 for AmB), similar to the effects seen with miltefosine and injected MA (*p* < 0.0001 in skin for both treatments). In contrast, the reductions in parasite loads in the liver showed somewhat lower significance (*p* = 0.044 for MA 30% in liver), but were still comparable to those observed with injected MA (*p* = 0.012 in liver) and more pronounced than those treated with miltefosine (*p* < 0.001 in liver).

Currently, the main treatments for CL involve pentavalent antimonials directly injected into the lesion, such as MA, which boasts an efficacy rate of over 70%, similar to that of intraperitoneally administered MA [11].

## 4. Discussion

Thus, Sepigel-based topical formulations are straightforward to prepare and offer a non-invasive route of administration that avoids injections, enhancing patient acceptance and tolerance. These formulations have been shown to be stable for at least six months, feature a biocompatible pH, spread easily, and exhibit no irritant effects on the skin, making them ideal for treating CL [14]. Consequently, several researchers have been exploring Sepigel formulations that incorporate various extracts or compounds for both medical and cosmetic applications. For example, Risaliti et al. [20] conducted a comparative study of Sepigel and a lipophilic cream in treating actinic keratosis, a precancerous condition caused by prolonged ultraviolet radiation exposure, characterized by scaly skin lesions. Their findings highlighted Sepigel’s superior therapeutic efficacy and patient compliance, attributed to its enhanced technological characteristics and stability. Additionally, a study focused on developing new O/W emulsions assessed their stability with active compounds, finding that Sepigel emulsions exhibited superior stability compared to other formulations containing active molecules [21]. Moreover, research by Potúcková et al. [22] noted a substantial enhancement in drug release with Sepigel 305 formulations, particularly with indomethacin as the active component.

Our therapeutic strategy offers two significant advantages related to the use of a gel for topical application: improving patient compliance and reducing toxicity profiles. Traditional treatments for CL often require intravenous, intramuscular, or intralesional administration, making them more invasive and less likely to be completed by patients. By offering a less invasive treatment method, our gel formulation could significantly enhance patient compliance, leading to better disease control from an epidemiological perspective [23]. Regarding toxicity, while the reference drug miltefosine and most existing leishmaniasis treatments exhibit significant systemic toxicity—including teratogenicity, nephrotoxicity, hepatotoxicity, and gastrointestinal issues [24]— our topical treatment could mitigate these risks by limiting drug absorption into the bloodstream, thus enhancing its safety for clinical use. Intriguingly, our research found a significant difference in parasite load between the miltefosine-treated group and our Sepigel-based treatment groups, particularly in the mice’s liver. Although the systemic nature of miltefosine may have contributed to its efficacy in reducing parasite load, our formulations containing 30% MA and AmB in Sepigel also effectively decreased this load, demonstrating their capability to prevent the infection from becoming visceral. These findings highlight the promising potential of our topical treatment in reducing toxicity and enhancing effectiveness in preventing the progression of the infection.

Regarding the efficacy of other similar studies assessing the *in vivo* activity of MA or AmB topical formulations, AmB has been applied in various methods for treating CL lesions caused by leishmaniasis. For example, it has been administered intravenously as a liposomal formulation, which is the most common method, and topically using micro-needling techniques [25]. AmB has also been used topically as a complex with cholesteryl sulfate or phospholipids in the presence of ethanol, achieving skin penetration and localized treatment of CL with very low total drug concentrations [26]. This study marks the first evaluation of AmB in a simple gel formulation for topical application. As for MA, while topical treatments typically involve injecting the drug directly into the lesion, our group is unique in having developed a simple gel formulation shown to be stable for at least six months and possessing optimal properties for CL treatment: biocompatible pH, easy spreadability, and no skin irritant effects [14].

However, although many similar studies have managed to reduce the parasite load more effectively than the reference treatment, few use miltefosine as the reference drug; instead, they often rely on older, more toxic drugs such as pentavalent antimonials or AmB [27]. Other authors have also investigated innovative approaches to drug delivery and pharmaceutical technology in the face of CL. Lalatsa et al. [28] explored the development of a nanoemulsion-based system for the targeted delivery of hydrophobic drugs such as buparvaquone, demonstrating enhanced bioavailability and therapeutic efficacy. In addition, de Sá et al. [29] explored the use of lipid nanoparticles in the delivery of poorly water-soluble drugs such as paromomycin, highlighting their potential to overcome challenges related to drug solubility and stability. Another group has created nanotransfersomal gel with the capability to retain and permeate the incorporated drugs like nitazoxanide through stratum corneum with promising results against cutaneous leishmaniasis [30].

In our study, a lesion reduction of 48% was observed two weeks post-treatment and 69% after four weeks using a 30% MA-Sepigel concentration. Conversely, AmB-Sepigel achieved a 72% lesion reduction after four weeks. When analyzing the parasite load in tissues, particularly skin, and using the untreated group as the baseline (100% of the parasite burden), we found that MA at 30% resulted in a lower parasite load (41%) compared to AmB (71%). Furthermore, despite some studies suggesting that treatment with intramuscular MA is more effective at curing cutaneous leishmaniasis than miltefosine [31], our *in vivo* study contradicts this finding. Oral miltefosine, at least after two weeks of treatment, showed better outcomes in reducing lesion appearance, size, and parasite load compared to injected MA, corroborating reports from other researchers [32].

This approach not only demonstrated a significant reduction in lesion size and parasite load in the *in vivo* study, but also showed improved patient compliance and a better safety profile compared to conventional systemic therapies. The results are especially promising for patients in endemic regions, where access to safer and more effective treatments is critical. Healthcare professionals and policy makers in these areas could benefit greatly from this study, as it paves the way for developing more accessible, more effective and tolerable treatment options that minimize systemic toxicity and improve overall adherence to CL treatment.

Formulations such as those studied in this work could reach the pharmaceutical industry market for veterinary use, which is much simpler than for human use, and commercialization could be achieved in the short term. For human treatment, however, it would be somewhat more costly, as Phase II and III clinical trials would be required. Although, it should be noted that the formulations evaluated in this work could be applied in a short period of time in clinical practice, since both the active ingredients and the excipients used are authorized for use, and would only involve formulation changes, and whose clinical trials would follow a process similar to that of new drugs, although shorter and less extensive, since this type of modification could affect the pharmacokinetics and pharmacodynamics of the compound. This is particularly relevant for skin treatments for leishmaniasis, a disease that affects not only humans but also many animals, such as dogs and cats.

## 5. Conclusions

Recent studies have demonstrated the promising potential of Sepigel-based formulations of MA or AmB as viable topical treatments for CL, particularly in combined drug studies. Notably, the highest tested concentration of MA (30%) showed superior effects on reducing both lesion size and parasite load across various tissues, with significant improvements observed in mice just two weeks into treatment. Furthermore, the development of a simple AmB gel marks a notable first in the literature and has shown high efficacy in treating CL. Sepigel-based treatments also simplify preparation and storage, and their topical administration avoids the common adverse effects associated with parenteral administration or intralesional injections, which are prevalent in current leishmaniasis treatments. In addition, its commercialization as a cutaneous treatment against CL for veterinary use could be quick and simple, and once achieved, its approval for human use could be obtained, as these require more complex clinical trials. These findings represent a significant advance toward developing more effective therapeutic approaches for this condition.

## Data Availability

The original contributions presented in the study are included in the article, further inquiries can be directed to the corresponding author/s.
